# The Hetero-Hexameric Nature of a Chloroplast AAA+ FtsH Protease Contributes to Its Thermodynamic Stability

**DOI:** 10.1371/journal.pone.0036008

**Published:** 2012-04-27

**Authors:** Ofer Moldavski, Olga Levin-Kravets, Tamar Ziv, Zach Adam, Gali Prag

**Affiliations:** 1 The Robert H. Smith Institute of Plant Sciences and Genetics in Agriculture, Hebrew University of Jerusalem, Rehovot, Israel; 2 Department of Biochemistry and Molecular Biology and the Institute for Structural Biology, The George S. Wise Faculty of Life Sciences, Tel Aviv University, Tel Aviv, Israel; 3 Department of Biology, Smoler Proteomics Center, Technion, Haifa, Israel; University of California – Davis, United States of America

## Abstract

FtsH is an evolutionary conserved membrane-bound metalloprotease complex. While in most prokaryotes FtsH is encoded by a single gene, multiple FtsH genes are found in eukaryotes. Genetic and biochemical data suggest that the *Arabidopsis* chloroplast FtsH is a hetero-hexamer. This raises the question why photosynthetic organisms require a heteromeric complex, whereas in most bacteria a homomeric one is sufficient. To gain structural information of the possible complexes, the *Arabidopsis* FtsH2 (type B) and FtsH5 (type A) were modeled. An *in silico* study with mixed models of FtsH2/5 suggests that heteromeric hexamer structure with ratio of 4∶2 is more likely to exists. Specifically, calculation of the buried surface area at the interfaces between neighboring subunits revealed that a hetero-complex should be thermodynamically more stable than a homo-hexamer, due to the presence of additional hydrophobic and hydrophilic interactions. To biochemically assess this model, we generated *Arabidopsis* transgenic plants, expressing epitope-tagged FtsH2 and immuno-purified the protein. Mass-spectrometry analysis showed that FtsH2 is associated with FtsH1, FtsH5 and FtsH8. Interestingly, we found that ‘type B’ subunits (FtsH2 and FtsH8) were 2–3 fold more abundant than ‘type A’ (FtsH1 and FtsH5). The biochemical data corroborate the *in silico* model and suggest that the thylakoid FtsH hexamer is composed of two ‘type A’ and four ‘type B’ subunits.

## Introduction

The AAA+ (ATPases associated with diverse cellular activities) super-family consists of protein complexes forming ring-shaped assemblies that convert chemical energy into motion or visa versa [1]. These highly conserved molecular machines carry out a vast number of cellular processes including cell division, cell differentiation, signal transduction, stress response and more [2]–[4]. Usually the AAA+ core consists of six homo or hetero protomers. In the case of proteolytic degradation, these macromolecule complexes tether protein unfolding to protease and thus enable the degradation of structural proteins. The mechanism of action of AAA+ proteases is intensively studied for several decades. Asymmetrical structure provides the possibility for a crosstalk within the assemblies [5]. Cycles of ATP binding, ATP hydrolysis, ADP bound state and ADP release in an asymmetrically circular manner has been demonstrated to play a pivotal role to achieve continues motion that promotes substrates unfolding [5]. The archaea proteasomal AAA+ complex, PAN, consists of six homomeric protomers. However, the eukaryotic proteasome consists of six different ATPase subunits that assembled into hetero-hexameric ring. Similarly, the prokaryotic AAA+ proteases including FtsH, Lon, HslU/V and the Clp proteins are assembled into homo-hexameric structures, while some of their eukaryotic orthologs present hetero-hexameric structures. Specifically, homologues of the bacterial FtsH protease exist in the organelles of prokaryotic origin, the mitochondria [6], [7] and chloroplasts [8], [9]. However, unlike most bacteria, these organelles possess multiple FtsH gene products. The mitochondrial FtsH homologues, which are conserved from yeast to humans, are encoded by three genes. The products of two of these form a heteromeric hexamer, designated mAAA, which is anchored to the inner membrane and faces the mitochondrial matrix. The third gene encodes a protein that assembles into a homomeric hexamer, designated iAAA, which is also attached to the inner membrane, but oriented towards the inter-membrane space [6], [7]. Similar to the *E. coli* enzyme, the yeast mAAA can associate with two negative regulators, designated prohibitins [10]. One can postulate that in eukaryotes, heteromeric structures increase the variability and specificity toward substrates and regulators.

Multiple FtsH genes exist also in photosynthetic organisms, from cyanobacteria to higher plants. The cyanobacterium *Synechocystis* has four genes, whereas higher plants such as *Arabidopsis* have as many as 12 genes encoding FtsH proteins, named FtsH1-FtsH12 [11]. Transient expression assays of GFP-fusions suggested that three of these, which are more similar to the yeast mitochondrial mAAA and iAAA, can be targeted to plant mitochondria, whereas the other nine, which are more similar to the cyanobacterial homologues, can be targeted to chloroplasts [12]. At least one species, FtsH11, can be targeted to both organelles [13]. Of the nine potentially targeted to chloroplasts, only four have been in fact identified in thylakoid membranes, namely FtsH1, FtsH2, FtsH5 and FtsH8 [8], [14]–[16]. This raises the possibility that the other homologues are either expressed only under certain conditions or in specific tissues, or alternatively, that they may be located in other sub-compartments of the chloroplast.

Analysis of two variegated *Arabidopsis* mutants, *var2* and *var1*, revealed that their respective pronounced and slight leaf-variegation phenotypes are caused by mutations in the genes encoding FtsH2 and FtsH5, respectively [17]–[19]. Further studies of these mutants allowed gaining insight into the physiological function of the thylakoid FtsH protease. First, the patchy phenotype - green sectors with normal chloroplasts that exist side by side with white sectors containing only proplastids, suggested that FtsH should be involved, either directly or indirectly, in the formation of thylakoids [17], [18], [20]. Second, *in vitro* and *in vivo* experiments provided evidences for the involvement of the FtsH protease in the repair of photosystem II (PSII) during photoinhibition and heat stress, as it participates in the degradation of oxidatively damaged reaction center protein D1 [19], [21]–[24]. In contrast, FtsH11 was shown to be essential for thermotolerance, but not for PSII repair [25]. It is also interesting to note that the role of the FtsH protease in PSII repair is not limited to higher plants, as a similar role was demonstrated in cyanobacteria as well [26], [27]

The aforementioned four gene products represent two sets of duplicated genes, which have been named ‘type A’ and ‘type B’. The former corresponds to FtsH1 and FtsH5, and the latter to FtsH2 and FtsH8 [28]. Over-expression studies in mutant backgrounds [16], [29], and analysis of single and double FtsH mutants [28], have suggested that whereas the duplicated genes within each type are redundant, the presence of both types is essential for the accumulation and function of the thylakoid FtsH complex. In terms of abundance, it was suggested that FtsH2 is the most abundant, followed by, in decreasing order, FtsH5, FtsH8 and FtsH1 [14]. Estimations based on immuno-blot analysis in a two-dimensional gel system suggested that the ratio between ‘type A’ and ‘type B’ subunits is 1∶2 (i.e., 2∶4 in a hexameric complex) [8], [28]. Although the genetic and biochemical evidence strongly support the presence of type A/B heteromeric complex, we regard the stoichiometry of its subunits only suggestive, as this is based on a single approach. Moreover, why photosynthetic organisms require a hetero-complex and not a homomeric one is totally unclear.

To get an insight why two types of subunits are essential for the FtsH complex to accumulate, we modeled the structures of the most abundant type A and B *Arabidopsis* proteins and showed that they are stabilized when form heteromeric complexes *in silico*. *In vitro* and *in vivo* biochemical studies were then performed to provide biological support for our *in silico* findings.

## Results

### Modeling of the thylakoid FtsH complex

**Figure 1:**
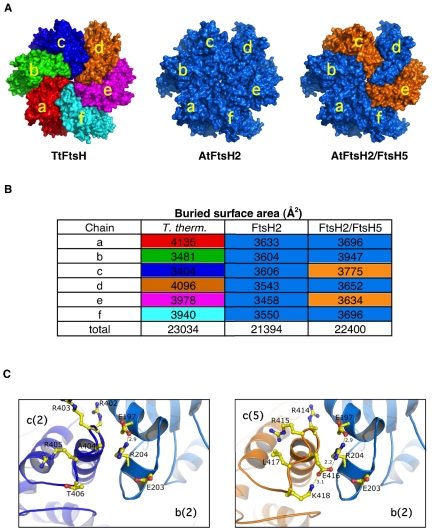
Models of the *Arabidopsis thaliana* FtsH complex. (**A**) TtFtsH (left), structure of *T. thermophilus* (PDB entry 2DHR) complex rendered as solvent accessible surface. Shown is the face of the protease side, with each chain in a different color. Models of the AtFtsH2 (middle) and AtFtsH2/5 rendered as TtFtsH. Chains of AtFtsH2 are colored in blue and chains of AtFtsH5 are colored in orange. (**B**) Summary of the calculated buried surface interfaces of the monomers in the *T. thermophilus*, AtFtsH2 and AtFtsH2/5 complexes. To facilitate the comparison, the values are color-coded as in *A*. (**C**) Zoom-in at the interface between chains *b* and *c*. The left and the right panels depict the homomeric (FtsH2) and the heteromeric (FtsH2/FtsH5) complexes, respectively. The picture in the left panel is centered at Arg-204 in chain *b* (marine blue), and its nearby residues in chain *c* (dark blue). Chain *c* of FtsH5 is colored in orange (like in 6A). Yellow dashed lines denote salt bridges; distances are indicated in Angstroms. Color code: oxygen – red; nitrogen – blue; carbon – yellow.

Several structures of the soluble domain of bacterial FtsH proteases were recently determined [30]–[33], all representing homo-hexameric structures. Interestingly, probably due to low stability of the hexameric complex, most of the AAA+ protein structures were determined as monomeric or sub oligomeric complexes. However, the one of *T. thermophilus* [32] (PDB id: 2DHR), was determined as full hexameric structure in which slight differences between monomers are presented. This structure allowed us to model the structure of the thylakoid FtsHs *in silico*. Sequence comparisons between the available Protein Data Bank (PDB) structures and the chloroplast FtsH2 and FtsH5 were made. We found that the closest homolog structure that was determined as a full homo hexmer is the one from *T. thermophilus* [32]. Alignment of FtsH2 and FtsH5 with this protein spans about 470 residues, with sequence identity of 51% and 53%, respectively. This homology level is adequate to perform sequence-based homology modeling with high confidence that the output model would assume the same fold. A recent study comparing common homology modeling algorithms showed that they all produce reasonable models when sequence identities are >30% [34]. It was recently shown that the yeast and human GAT domains that form the core of the ESCRT-0 complex present nearly identical crystal structures (with RMSD of 1.58 Å) at only 5% a.a. sequence identity [35], [36]. We thus modeled the structures of both FtsH2 and FtsH5 based on the structure of the *T. thermophilus* protease. As shown in Figure 1A and, the FtsH2 hexamer closely resembles the bacterial FtsH hexamer. One significant structural parameter that shed light on the affinity between subunits and thus on the stability of the protein complexes is the area of surface that is buried in the protein-protein interface. Chothia and Janin [37] demonstrated that the association between proteins described by Van der Waals' contacts, electrostatic forces and hydrogen bonds could account for their stability. Using buried surface area calculations developed by Levitt and Chothia [38] and analyses of the hydrophilic interactions, they showed that the buried surface area plays the major stabilization factor in protein-protein association. Therefore, from several structural parameters that could be attributed to the total stability of protein complexes, the buried surface area presents the highest correlation between calculated and measured data [39]. Moreover, such calculation reduces risks of over estimation in assessment of a molecular model derived from homology modeling, since it fine analyzes the structure to a lesser extent. We therefore calculated the buried surface areas between adjacent monomers in the homohexameric structures (these values represent the sum of interfaces of one monomer with its two neighbors). Although the bacterial homohexamer has a six fold pseudo-symmetry, slight structural differences between its monomers are translated into different values of the buried surface areas, ranging from 3404 to 4135 Å^2^ (Figure 1B). Similarly, the modeled FtsH2 homohexamer also shows variability in the buried area of each monomer, but it appears less stable than the bacterial protease, with a total of 1640 Å^2^ less buried surface area.

The high similarity between FtsH2 and FtsH5 permitted substitution of protein chains in the homomeric structures, to create a mixed structure of FtsH2/FtsH5. We performed systematic substitutions of monomers between FtsH2 and FtsH5, followed by energy minimization and careful inspection of the structures to ensure that there are no structural clashes. Comparing the buried surface areas between the mixed complexes and the homo-complex of FtsH2, we found that most of these permutations substantially decreased the amount of buried area at both sides of the monomer. Interestingly however, when we substituted one of the *a*, *c* or *e* chains of FtsH2 with the corresponding chain of FtsH5, we found a substantial increase in the buried surface area, amounting to 183, 160 and 182 Å^2^, respectively (Figure S2). Moreover, a net positive effect on the other subunits of the complex resulted in an overall increase of 484, 459 and 574 Å^2^ compared with the homomeric FtsH2 complex.

Modeling the substitution of two FtsH2 subunits with two FtsH5 ones shows that the overall structure of this mixed complex, in which chains *c* and *e* were substituted, is similar to the homomeric complex of *T. thermophiles*. Moreover, the buried surface area of these subunits increases by 169 and 175 Å^2^ respectively, with this substitution (Figure 1B). The other four subunits are also positively affected by these substitutions, resulting in a net increase of 1006 Å^2^ in the buried surface area compared with the homomeric FtsH2 hexamer. Thus, it appears that although ‘type B’ subunits are more abundant than ‘type A’ ones, the formation of a mixed complex containing both types of subunits is thermodynamically favored over the formation of a homomeric hexamer composed of ‘type B’ subunits only.

A similar effect was observed when all three chains were substituted, leading to an increase of 1067 Å^2^ in the buried surface area (Figure S1). However, the total buried area here is only slightly higher than in the *c*+*e* substitution (22461 Å^2^ vs. 22400 Å^2^, respectively). Therefore, from a thermodynamic point of view only, equal representation of both subunits in the hexamer is a possibility that should not be ruled out.

The increase in buried surface area observed in the heteromeric complex can be attributed to new hydrogen bonds and salt bridges. Zooming into the interface between chains *b* and *c* provides one such example (Figure 1C). Based on our models, the NH_2_ group of Arg204 in chain *b* makes a salt bridge with OE1 of Glu197 of the same chain. In the case of a homomeric complex, Arg204 does not form additional bonds with its neighbor residues in chain *c*. However, substitution of the FtsH2 chain *c* with the one of FtsH5 results in an alteration of three residues at the nearby interface (Ala-Arg-Thr in positions 404–406 to Glu-Leu-Lys in positions 416–418). The substitution of Ala404 with Glu416 allows the free NH1 group of Arg204 to form a new salt bridge with OE1 of Glu416 in chain *c*. Moreover, the orientation of Glu416 (of FtsH5 chain *c*) toward Arg204 (of FtsH2 chain *b*) is locked by additional salt bridge donated by Lys418. Our model suggests that the substitution, of the FtsH2 chain *c* with the one of FtsH5, not only increases the buried surface area and thus allowing more hydrophobic interactions, but also promotes the formation of a new network of hydrogen bonds and salt bridges. Careful examination of these networks shows that there are 76 hydrophilic interactions in the homomeric complex and 81 in the heteromeric complex. Specifically, the chain exchanges led to a gain of 8 salt bridges and a loss of 3 hydrogen bonds. This is equivalent to additional decrease in the free energy by about 4.0 Kcal/mol.

### Generation and characterization of FtsH2-HA transgenic plants

**Figure 2:**
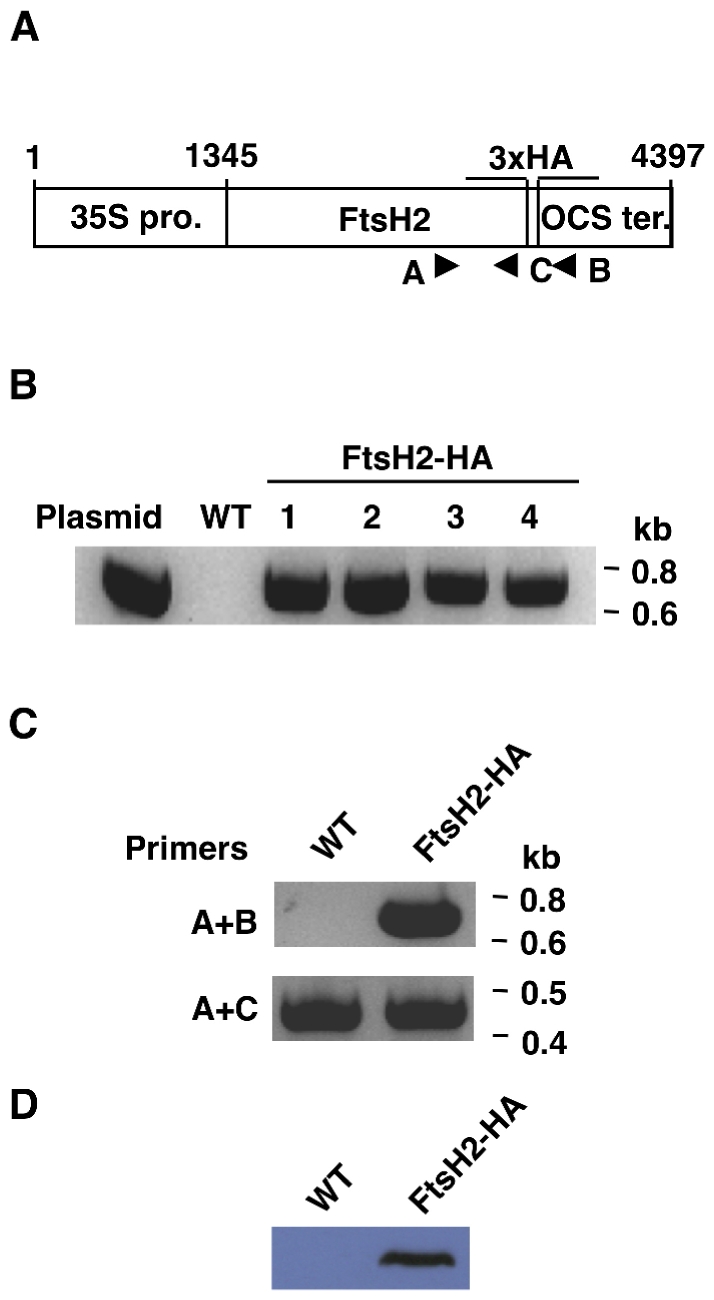
Characterization of transgenic plants expressing epitope-tagged FtsH2. (**A**) Schematic representation of the HA-tagged FtsH2 construct. A 3×HA tag sequence was cloned in-frame to the FtsH2 cDNA 3′ end. The construct was cloned between the 35S constitutive promoter and the OCS terminator. Arrowheads correspond to primers used in further analyses. (**B**) PCR on genomic DNA of kanamycin-resistant transgenic plants. Primers A and B (shown in *A*) were used to distinguish between WT and transgenic plants. (**C**) Qualitative RT-PCR on total RNA. Primers A and B were used to detect FtsH2-HA transcripts. Primers A and C were used to detect the native FtsH2 transcript. All other transgenic seedlings demonstrated a similar behavior. (**D**) Immuno-blot analysis of total protein from FtsH2-HA and WT plants. The blot was reacted with an HA antibody. Load is 10 µg chlorophyll per lane. All other transgenic seedlings demonstrated a similar behavior.

For *in vivo* assessment of our *in silico* data, we generated plants that expresses an epitope-tagged FtsH2. We chose to fuse three copies of the HA tag in tandem to its C-terminus (Figure 2A), as crystal structures of the soluble domain of bacterial FtsHs suggest that this end is relatively free [31], [32]. In addition, fusion to this end avoids possible complications due to ambiguity regarding the processing site of the chloroplast-targeting sequence. Transformants were selected on Kanamycin, and the presence or lack of the transgene in transformed and WT plants, respectively, was confirmed by PCR (Figure 2B). Accordingly, we verified its expression in the transformed plants by RT-PCR (Figure 2C). Finally, we tested whether the transgenic transcript was translated. Antibodies against the HA tag cross-reacted with a band of the expected size (ca. 70 kD) in the transgenic plants whereas no cross-reaction was observed in WT samples (Figure 2D).

**Figure 3:**
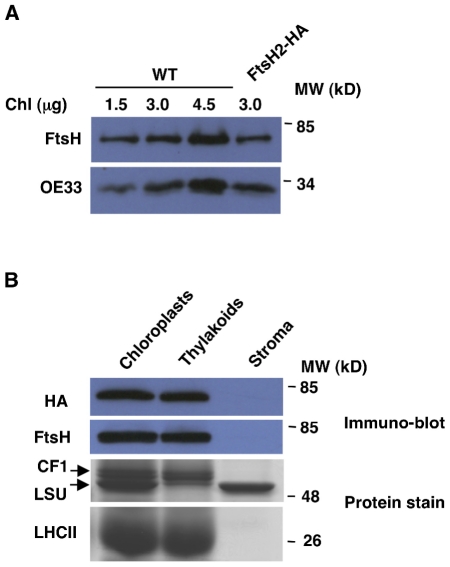
Abundance and localization of the FtsH2-HA protein. (**A**) Immuno-blot analysis of total protein, extracted from transgenic and WT plants. Antibodies used are indicated on the left. (**B**) Intact chloroplasts were isolated from FtsH2-HA plants and fractionated into thylakoids and stroma. Samples were separated by SDS-PAGE, and gels were either stained by Coomassie blue or blotted onto membranes and reacted with anti-HA and anti-FtsH antibodies. All samples contained equivalents of 3 µg chlorophyll, except the HA blot, which contained equivalents of 10 µg chlorophyll. The antibodies used, or the location of specific proteins on the stained gels, are indicated on the left.

The transgenic FtsH2-HA plants were visually indistinguishable from WT, however we suspected that they might have elevated levels of FtsH2 compared to WT plants, as the tagged FtsH2 was expressed under the control of a strong constitutive promoter. Thus, we compared the level of FtsH2 in WT and transgenic plants. As shown in Figure 3A, the transgenic plants accumulated similar, or even slightly lower, levels of FtsH2. This finding is consistent with our previous unsuccessful attempts to over-express FtsH2 in plants, and suggests that there may be an upper limit for the amount of FtsH protease that can accumulate in thylakoid membranes.

To test whether the C-terminal tag of FtsH2 had any affect on import into the chloroplast and sorting within it, we isolated intact chloroplasts from transgenic plants, fractionated them into thylakoids and stroma, and subjected them to SDS-PAGE and immuno-blot analyses. The tagged FtsH2 indeed accumulated in the thylakoids, similar to native FtsH and other thylakoid markers such as subunits of the ATP-synthase (CF1) and the antenna of photosystem II (LHCII), and unlike the stromal marker, the large subunit of Rubisco (Figure 3B).

### Purification of FtsH2-HA

**Figure 4:**
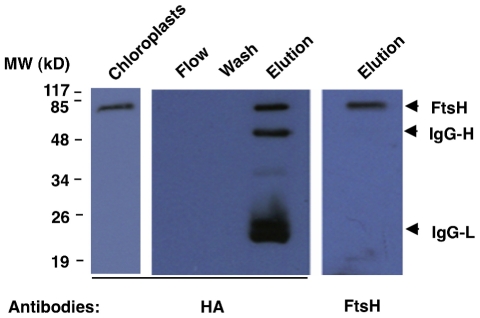
Immuno-purification of FtsH2-HA. Intact chloroplasts were first isolated from FtsH2-HA plants, then lysed and solubilized with 1% β-DM. Anti-HA agarose conjugate was added and washed extensively, before elution with 2× sample buffer. Samples from the different steps were resolved by SDS-PAGE, blotted and reacted with HA and FtsH antibodies.

To purify the tagged FtsH2, we developed a one-step purification protocol. Prior to the purification step itself, intact chloroplasts were isolated on a Percoll gradient, and then solubilized with the mild detergent n-dodecyl β-D-maltoside (final concentration of 1% β-DM for 15 min on ice). After clearing the solubilized material by centrifugation, it was mixed with an HA antibody immobilized onto agarose beads. Unbound material was removed, and the matrix was washed with excess buffer. Finally, the bound material was harshly eluted from the matrix by two-fold concentrated protein sample buffer. Immuno-blot analysis of samples from the different stages of the protocol revealed that proteins cross-reacting with anti-HA were found only in the chloroplast crude material, as a single band, and in the eluted material, as multiple bands (Figure 4). To determine the nature of these bands, the membrane was stripped and re-probed with antibodies against FtsH, thus ascertaining that the 70-kD region contains FtsH protein(s). The smaller bands that were detected by anti-HA were consistent with the sizes of the heavy and light chains of IgG. This is in agreement with the manufacturer's product statement that harsh elution releases some HA antibodies from the agarose matrix.

### Mass spectrometry analysis of purified FtsH2

**Figure 5:**
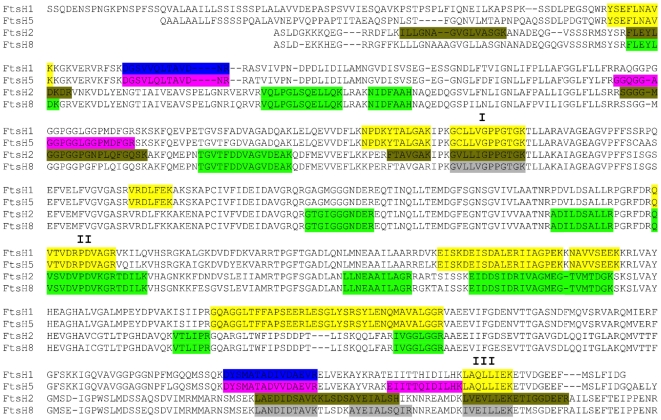
Mass spectrometry analysis of purified FtsH2-HA. Alignment of mature FtsH1, FtsH5, FtsH2 and FtsH8. Highlighted are peptides identified by MS. Yellow, peptides conserved between FtsH1 and FtsH5; green, peptides conserved between FtsH2 and FtsH8; blue, peptides specific to FtsH1; purple, peptides specific to FtsH5; olive, peptides specific to FtsH2; gray, peptides specific to FtsH8. Red Roman numerals indicate peptide groups used for quantification.

We first performed an MS analysis of a gel slice that corresponds to the band that cross-reacted with the HA and FtsH antibodies (Figure 4). As expected, tryptic peptides diagnostic of FtsH2 were recovered. Nine of these were unique to FtsH2 (Figure 5, Table S1), while another thirteen represent sequences that are fully conserved between FtsH2 and FtsH8. In addition, four peptides specific to FtsH8, but not to FtsH2, were identified. Moreover, eleven tryptic peptides derived from conserved sequences of FtsH1 and FtsH5 were identified, as well as two and four specific peptides from these orthologs, respectively (Figure 5, Table S1). Similar results were obtained in an independent duplicate experiment (Figure S2). Altogether, these results demonstrate that in thylakoids, FtsH forms a heteromeric complex comprised of both ‘type A’ (FtsH1 and FtsH5) and ‘type B’ (FtsH2 and FtsH8) subunits.

**Figure 6:**
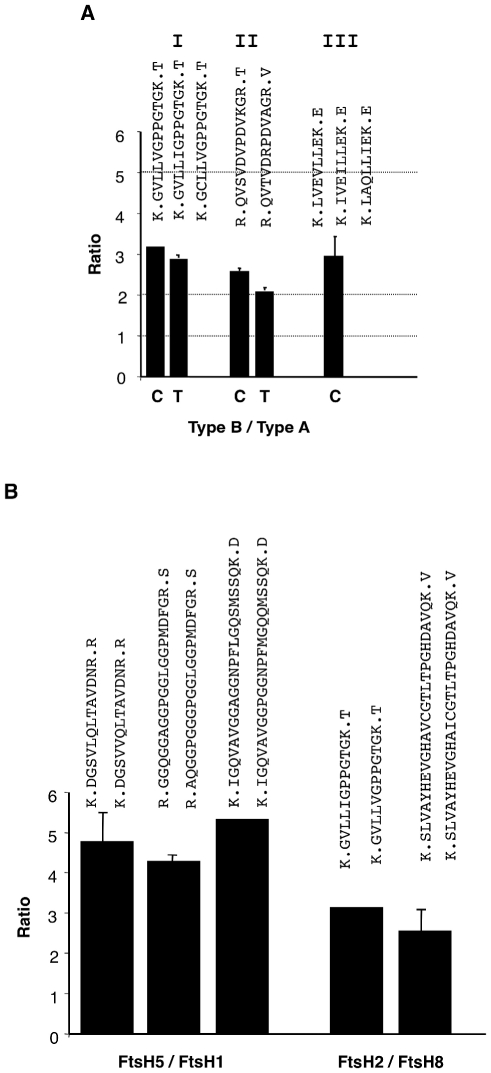
Relative abundance of FtsH subunits. The abundance of FtsH subunits was deduced from the peak area of specific peptides identified in the MS analysis. (**A**) Ratios between ‘type B’ and ‘type A’ subunits in isolated complexes (C) and intact thylakoids (T). The Roman numerals correspond to those indicated in Figure 4. The horizontal dashed lines represent possible ratios within the hexamer. (**B**) Ratios between the products of duplicated genes within a type. The sequences of the peptides used in the analysis are indicated above the bars. Values are averages ±s.d. of 2–4 replicates.

To gain insight into the stoichiometry between ‘type A’ and ‘type B’ subunits within the isolated FtsH complex, we selected three groups of peptides (see Figure 5) and quantified their relative abundance by compiling their peak areas from the MS data. The selection criteria for peptides were similar length and minimal sequence variation. Normalizing to the level of the ‘type A’ peptides, we observed that ‘type B’ peptides were approximately 3-fold more abundant than ‘type A’ peptides in the three comparisons we made (Figure 6A, ‘C’ columns).

Reasoning that a normal ratio of subunits within the complex could be distorted by the strong constitutive promoter controlling the expression of the tagged FtsH2, we decided to evaluate the ratio between FtsH subunits in WT plants. For this purpose, we subjected isolated thylakoids to SDS-PAGE, excised a piece of gel from the 70-kD region, and analyzed it by MS, similar to the analysis performed on the isolated complex. Given the greater amount of material in this analysis compared with that of the isolated FtsH complex, we obtained a better coverage of peptides, which included those used for the aforementioned quantification. However, group III of peptides included a mixture of overlapping peptides of different lengths, which prevented their reliable quantification. Analysis of the first two groups revealed a tendency similar to that observed with the isolated complex; ‘type B’ subunits were 2–3 fold more abundant than ‘type A’ (Figure 6A, ‘T’ columns). Nevertheless, in both groups of peptides, the ratio was slightly though significantly lower in thylakoids compared with the isolated complex.

As all characterized FtsH proteases, including the thylakoid one, form hexamers, three distinct ratios between ‘type B’ and ‘type A’ subunits can be possible: 5∶1, 4∶2 or 3∶3. The ratio found in this analysis suggests that the thylakoid FtsH protease is composed of four ‘type B’ subunits and two ‘type A’ subunits, which is in agreement with the quantification of FtsH spots on a 2D gel, identified by immunoblot [14] and with our *in silico* modeling data. Nevertheless, we cannot exclude the possibility of variable ratios within a complex, with some hexamers containing two ‘type A’ subunits and some only one, and that the ratio obtained here may represents an average composition rather than a set stoichiometry. Alternatively, this non-stoichiometric overrepresentation of ‘type B’ subunits may result from the presence of some monomers or partially assembled complexes, whose presence was recently demonstrated in spinach thylakoids [Yoshioka et al. 2010].

Our MS data also allowed us to determine the ratios between the products of duplicated genes within the FtsH complex, though we do not expect these to have functional significance. Analysis of three sets of peptides revealed that FtsH5 is 4.5–5 fold more abundant than FtsH1 (Figure 6B). Similarly, analysis of two suitable sets suggested that FtsH2 is 2.5–3 more abundant than FtsH8. These results are consistent with our previous finding that FtsH2 is more abundant than FtsH8, and FtsH5 is more abundant than FtsH1 [14], and therefore meets our *in silico* modeling and corroborates the current MS quantification analysis.

To assess whether the purified FtsH complex contained other non-FtsH partners, similar to the *E. coli* and the yeast homologous complexes, we subjected the entire eluted complex to MS analysis. For this purpose, the material eluted from the affinity matrix was separated by SDS-PAGE, and the gel was sliced into five pieces, each of which was subjected to MS analysis. Peptides recovered from this analysis corresponded to the α and ß subunits of ATP synthase, the A1 protein of the PSI reaction center, the Lhcb2 protein of the PSII antenna, the CP43 subunit of PSII, and the large subunit of Rubisco (Table S2). However, the amounts of these were 5% or less compared with the amounts of FtsH subunits, suggesting that these represented contamination by abundant proteins rather than components of the FtsH complex.

## Discussion

The existence of multiple FtsH genes in photosynthetic organisms, compared with the single FtsH gene found in most bacteria, has been enigmatic. We have previously observed that double-mutant plants lacking either ‘type A’ or ‘type B’ subunits are albino - thus able to grow only heterotrophically on sucrose, and that they do not accumulate the supposedly expressed other FtsH subunits [28]. This proposes that chloroplast FtsH is either unable to assemble into an all-A- or all-B-type complex, or that a homo-complex is unstable, and hence raises the obvious question of how this complex differs from the bacterial FtsH hexamer, which is composed of six identical subunits. Modeling of the thylakoid FtsH presented in Figure 1 and Figure S1 allowed us to gain insight into this question. It has been suggested that, in terms of energy, one square Angstrom of buried surface area is equal to approximately −16 calories [39], [40]. Thus, a homomeric FtsH2 complex should have a total of 26 Kcal more than the complex in *T. thermophilus*. In contrast, the introduction of two or three FtsH5 subunits into the complex should decrease its free energy by approximately 16–20 Kcal, making it thermodynamically more stable. Thus, the *in vivo* situation, where ‘type B’ subunits are twice more abundant than ‘type A’, would favor the formation of a hetero-complex over a homomeric complex of ‘type B’.

As already mentioned, the modeling of the chloroplast FtsH was done only on its soluble domain, because the structure of the membrane anchors is unknown in any organism. It is expected that these hydrophobic domains will also contribute to the stability of the complex, but at this time it is impossible to assess to what extent. Other non-FtsH factors are also expected to affect the *in vivo* stability of the FtsH complex. These may include other thylakoid proteins, lipids, cofactors, *etc*.

To gain insight into the composition of the FtsH protease operating in the thylakoid membranes of higher plants, we generated plants expressing HA-tagged FtsH2 and analyzed these transgenic plants. To facilitate detection and purification of the tagged protein, we expressed it under the control of a strong constitutive promoter. Detection with a HA-specific antibody was feasible, but we did not observe over-expression of FtsH when compared to WT (Figure 3). We suspect that there may be an upper limit to the amount of FtsH protease that can accumulate in the thylakoid membrane. This is interesting especially in light of the fact that reduced levels of FtsH lead to a variegated phenotype, and the suggestion that a fine balance between protein synthesis and degradation is essential for normal chloroplast development and maintenance, as revealed by suppressor analysis of the variegated phenotype [41], [42].

The MS analysis of immuno-purified FtsH2-HA, which recovered peptides diagnostic of FtsH1, FtsH2, FtsH5 and FtsH8, provides the first direct evidence for the interaction between FtsH2 and each one of the three other isomers. Circumstantial evidence for the presence of a heteromeric complex have been provided previously: mutants devoid of either FtsH2 or FtsH5 have lower levels of the other subunit; these two proteins also co-migrate on sucrose gradients and gel filtration columns [12]. The results of this work provide proof for the presence of each one of the other orthologs in the same complex with FtsH2.

The MS analysis also allowed obtaining clues into the stoichiometry of the different subunits within the heterocomplex. The peak area associated with specific peptides of similar length and sequence are correlated with their relative abundance. Analysis of three different sets of peptides from the purified tagged complex revealed that ‘type B’ subunits are two to three folds more abundant than ‘type A’. Slightly lower ratios, somewhat closer to two, were obtained when isolated thylakoid membranes were subjected to the same analysis. This suggests that FtsH2 may be over-represented in the transgenic lines due to its expression under the control of the 35S promoter. If this is indeed the case, these lines may contain complexes with variable stoichiometries that deviate from the one found in WT plants, and/or monomeric or partially assembled FtsH2 complexes, as was recently observed [Yohioka et al. 2010].

Assuming that the thylakoid FtsH heterohexamer consists of a fixed ‘type B’ to ‘type A’ subunit ratio, this ratio can be 1, 2 or 5 (see Figure 6A), corresponding to 3, 2 or 1 ‘type A’ subunits per hexamer, respectively (or the converse numbers if ‘type A’ subunits are more abundant than ‘type B’). Thus, quantification of the homologous peptides suggests that the FtsH complex is most likely composed of four ‘type B’ subunits and two ‘type A’ ones. Interestingly, this ratio is consistent with a ratio deduced from immunoblot analysis of a 2D gel with an antibody made against a peptide representing a conserved sequence found in all four subunits [14], [28] and validates our modeling study. Nevertheless, it should be emphasized that representation of the redundant subunits within a type is determined by their differential abundance.

An additional piece of information that could be extracted from our MS data is the relative abundance of subunits within a type. The ratios between FtsH1 and FtsH5, and FtsH2 and FtsH8 are not expected to have any functional significance, as the duplicated genes are redundant, and null mutants of FtsH1 and FtsH8 are indistinguishable from WT [16], [28], [29]. Nevertheless, we selected several peptides for determination of these ratios and found that FtsH5 is four-five times more abundant than FtsH1, and that FtsH2 is two-three times more abundant than FtsH8 (Figure 6B). The different levels may reflect differences in the strength of the respective endogenous promoters. It should be noted that consistent with the essentiality of the two types of subunits, but inessentiality of the gene duplication, is the observation that the two types are found in all sequenced genomes of photosynthetic organisms. However, in certain genomes, e.g., in the green alga *Chlamydomonas reinhardtii*, there is only one gene for each type [43].

The thylakoid FtsH complex is not the only heteromeric FtsH complex. As mentioned above, the mitochondrial mAAA protease is also composed of two homologous subunits [6], [7]. However, in contrast to the thylakoid complex, each of these two subunits are found in the hexamer in three copies [44], [45], making the thylakoid FtsH the first known case of uneven stoichiometry within the otherwise pseudo-symmetric complex. It remains to be seen whether this asymmetry has any functional implication. Moreover, the inferred increase in stability by hetero-hexamerization raises the possibility that the mAAA protease, as well as other similar complexes, may also be stabilized by their heteromeric nature and perhaps hints at the evolutionary driving force behind multiplication of FtsH genes.

Although in general crystal structures provide a static view of a given protein, in some cases where several protomers are crystallized, the structure of these subunits may provide insight into conformational changes. The structure of *T. thermophiles* FtsH is such an example. An interesting feature revealed during the calculation of buried surface areas in the *T. thermophilus* pseudo-symmetric complex was that the buried surface area for each chain was different. Slight structural conformational changes reflected by the differences in the structure of each chain, even though they all possess a fully identical sequence and bind the same ligand (ADP). Not only *T. thermophilus* presents structural and buried surface changes. The structure of *Thermotoga maritima* FtsH (PDB entry 2CE7) similarly presents variations of up to 16% between its monomers buried interfaces (see Table S3). Do pseudo-symmetric structures within the AAA+ family always present such variation? The crystal structure of the ND1 inactive AAA+ of P97 for example presents nearly identical interfaces between the homomers with maximal variation of 1.3% (range from 2,250 Å^2^ to 2,280 Å^2^; see Table S3). The latter may suggest that these structural changes reflect biological function rather than crystallographic constrains. It is possible that these minor changes represent a manifestation of the dynamic nature of AAA+ proteins as was recently showed by Goldberg and co-workers. [5] In accordance with this observation, substitution of FtsH2 chains with FtsH5 ones resulted not only in a change of the surface area at a certain position of the complex, but also in an allosteric effect in the other chains. Based on the current models, further experimentation, using a site-directed mutagenesis approach, will be useful in determining the contribution of specific residues or sequences to stabilization or destabilization of the thylakoid FtsH complex.

## Materials and Methods

### Model building and buried area calculation

The At-FtsH2/5 protein sequences were used as query for BLAST against the Protein Data Bank (PDB) and revealed that the FtsH from *T. thermophilus* (entry 2DHR), whose hexameric structure has been determined [32], was the closest homolog for both AtFtsH2 and AtFtsH5. The GENO3D software [46] was used for modeling the structures of AtFtsH2 and AtFtsH5. Each of the hexmeric protein chains of 2DHR was used to model homo hexameric structures of both AtFtsH2 and AtFtsH5. The monomers were assembled into an oligomeric complex by combining the files, and furtherer minimized using Refmac5 [47]. Finally, the oligomeric structures were visually inspected by PyMol and Rasmol [48], to ensure that there were no clashes in the final models. Structural figures were made using PyMol. We replaced chains between the homomeric structures to yield a mixed AtFtsH2 and AtFtsH5 complexes and these structures were minimized and inspected as described above. Solvent accessibility surface area was calculated using the areaimol algorithm of the CCP4 with water molecule radius of 1.4 Å [49]. The buried interfaces between adjacent protein monomers were calculated for each neighbor pairs. Permutations of chains between AtFtsH2 and AtFtsH5 were generated by superimposition followed by minimization as described above, and the buried areas were recalculated accordingly.

### Molecular Cloning

Total RNA was extracted from WT plants using standard protocols and converted to cDNA using SuperScript™ II Reverse Transcriptase (Invitrogen). The FtsH2 cDNA was amplified using the forward primer TACTAGTAATCGATACAGATGG and the reverse primer GCATTTAAATACAGCAGCTGGTGTTGGTGCT. A sequence encoding three copies of the HA epitope (3×HA) was amplified using the forward primer GCATTTAAATATTACCCATACGATGTTCCTGA and the reverse primer TATCATGCGATCATAGGCGTCACTAGTAT. Both PCR products were cloned together to form a continuous ORF, with the 3×HA at the 3′ end of FtsH2, in the binary vector pART27, in between the constitutive 35S promoter and the Ocs terminator (see Figure 1A).

### Plant growth, transformation and selection

*Arabidopsis thaliana* plants from the ecotype Columbia represent wild type (WT) and the background for the transgenic plants. Plants were grown in Kekkila peat (Finland) in controlled growth chambers, in short (10 h light/14 h dark) or long day (16 h light/8 h dark) at 22°C. RH was 70% and light intensity was 100 µE m^−2^ s^−1^. Transformation of WT plants was done by the flower dip method as previously described [50]. Seeds were sterilized and sown on MS media containing 25 µg/ml Kanamycin (Duchefa) for selection of transformants. The presence of the construct was verified by PCR on genomic DNA, isolated as previously described [28], using a forward primer from FtsH2 (GTGATTCCGAGGTGACTACTGG) and a reverse primer from the Ocs terminator (ATAGGCGTCTCGCATATCTC). RNA was extracted from WT and transgenic plants and converted to cDNA as above. The same primers were used to verify mRNA accumulation of the transgenic construct. The above forward primer and a reverse primer (GCATTTAAATACAGCAGCTGGTGTTGGTGCT) from the 3′ of FtsH2 were used as control for cDNA intactness.

### Protein extraction, gel electrophoresis and immuno-blot analysis

Protein extracts were obtained by grinding 100 mg of green leaves in 300 µl of 4× sample buffer (0.2 M Tris-HCl pH 6.8, 5 M urea, 8% [w/v] SDS, 40% [v/v] glycerol, and 20% β-mercaptoethanol). Blots were reacted with the following antibodies: monoclonal anti-HA (Sigma) at 1∶1,000 dilution; anti-FtsH or anti-OE33 [20] at 1∶5,000 or 1∶15,000 dilutions, respectively. Secondary anti-mouse HRP antibody and anti-rabbit HRP antibody were used at 1∶5,000 and 1∶10,000 dilution, respectively. All blots were developed using ECL solution (1.25 mM luminol, 0.2 mM paracumaric acid, 0.00915% H_2_O_2_, 100 mM Tris-HCl, pH 8.0).

### FtsH2 co-immunoprecipitation

Chloroplasts were isolated as described [51]. Intact chloroplasts pellet was resuspended with PBS (137 mM NaCl, 2.7 mM KCl, 10 mM Na_2_HPO_4_, 2 mM KH_2_PO_4_, pH 7.4) and protease inhibitor (Mini-complete, Roche). Protein concentration was adjusted with PBS to 8 µg/µl, and equal volume of PBS containing 2% n-dodecyl β-D-maltoside (β-DM) (Sigma) was added in order to solubilize the chloroplast membranes. After 15 min incubation on ice, insoluble membranes and debris were spun down at 137,000 g for 10 min. 100 µl of anti-HA agarose conjugate (Sigma) was added to the supernatant and incubated for one hour at 4°C. The anti-HA agarose matrix was washed four times with 1 ml of washing buffer (PBS containing 0.1% β-DM), and proteins were eluted with 100 µl of 2× sample buffer.

### Mass spectrometry

The proteins in gel slices were reduced with 4 mM DTT, modified with 15 mM iodoacetamide and trypsinized (modified trypsin, Promega) at a 1∶10 (w/v) enzyme-to-substrate ratio. The resulting tryptic peptides were resolved by reverse-phase chromatography on 0.075×200 mm fused silica capillaries (J&W) packed with Reprosil reversed phase material (Dr Maisch GmbH, Germany). The peptides were eluted with linear 65-minutes gradients of 5 to 45% acetonitrile and 15 minutes at 95% acetonitrile with 0.1% formic acid in water at a flow rate of 0.25 µl/min. Mass spectrometry was performed by an ion-trap mass spectrometer (Orbitrap, Thermo) in a positive mode using repetitively full MS scan followed by collision induced dissociation (CID) of the seven most dominant ions selected from the first MS scan. The mass spectrometry data was clustered and analyzed using Sequest and Pep-Miner [52], searching against the *Arabidopsis* database. In order to compare between the quantities of the different isoforms, several unique peptides were used. As the peak sequence can alter the peak intensity, we chose similar peptides with minimal sequence changes. The MS peaks of those peptides were detected using the ICIS algorithm and their areas were calculated with a mass tolerance of 0.01 Da. The ratio between the peak areas of similar peptides from different isoforms gives a semi-quantitative indication of the ratio between the isoforms.

## Supporting Information

Figure S1 Peptides Identified in a duplicate mass-spectrometry analysis of purified FtsH2-HA.
(EPS)

Figure S2 Models of mixed FtsH2/FtsH5 complexes. Color codes are as in figure 1. The numbers are the calculated buried surface interfaces of each monomer.
(EPS)

Table S1 Mass-spectrometry data of FtsH peptides identified in the analysis of purified FtsH2-HA.
(EPS)

Table S2 Mass-spectrometry analysis of chloroplast proteins co-purifying with FtsH2-HA.
(PDF)

Table S3 Buried surface areas (in Å) in *T. maritima* FtsH and Human P97-ND1 (PDB accessions 2CE7 and 1S3S, respectively).
(EPS)
