# Supplementary material for: The Hetero-Hexameric Nature of a Chloroplast AAA+ FtsH Protease Contributes to Its Thermodynamic Stability
Source: PLoS One. 2012 Apr 27;7(4):e36008. doi: 10.1371/journal.pone.0036008 (PMC3339871; doi:10.1371/journal.pone.0036008)
Supplement: Table S2 — Mass-spectrometry analysis of chloroplast proteins co-purifying with FtsH2-HA. (PDF) [file pone.0036008.s004.pdf]

Supplemental Table 2. MS Analysis of Chloroplast Proteins Co-Purifying with FtsH2-HA.

Seq 20187 - #1 - No Identification

Seq 20188 - #2

Serial#	Gi	Protein Name	Comment	Identified peptides		> Score	> Mass	Sequence	Charge	xCorr		Coverage
1	30684767	VAR2 (VARIEGATED 2); ATP-dependent peptidase/ ATPase/ metallopeptidase/ zinc ion binding [Arabidopsi ...		11								18%
						> Score	> Mass	Sequence	Charge	xCorr		
					1	95	1396.791	ADILDSALLRPGR	3	1.8472		
					2	75	962.4426	EIDDSIDR	2	1.7605		
					3	76	1546.811	LSDSAYEIALSHIK				
					4	88	1032.471	GTGIGGGNDER	2	2.6868		
					5	90	1060.552	LAEDIDSAVK	2	2.5473		
					6	87	1188.647	LAEDIDSAVKK	2	2.8219		
					7	82	1198.61	FLEYLDKDR	2	1.8303		
					8	88	1408.681	IVAGMEGTVMTDGK	2	3.6813		
					9	94	1424.676	IVAGM(+15.995)EGTVMTDGK	2	3.6813		
					10	91	1424.676	IVAGMEGTVM(+15.995)TDGK	2	3.6813		
					11	82	1440.671	IVAGM(+15.995)EGTVM(+15.995)TDGK	2	3.6813		
					12	90	1452.842	VQLPGLSQELLQK	2	3.0845		
					13	83	1516.829	EAMDKLVEVLLEK	2	2.5205		
					14	82	1902.876	SGGGM(+15.995)GGPGGPGNPLQFGQSK				
Serial#	Gi	Protein Name	Comment	Identified peptides								Coverage
2	18422193	VAR1 (VARIEGATED 1); ATP-dependent peptidase/ ATPase/ metallopeptidase [Arabidopsis thaliana]		5								7%
	18402995	FTSH1 (FtsH protease 1); ATP-dependent peptidase/ ATPase/ metallopeptidase [Arabidopsis thaliana]										7%
	1483215	chloroplast FtsH protease [Arabidopsis thaliana]				> Score	> Mass	Sequence	Charge	xCorr		7%
					1	79	875.4471	NAVVSEEK	2	1.5375		
					2	80	924.4788	LESGLYSR	2	1.9012		
					3	77	1198.647	YSEFLNAVKK	2	2.1127		
					4	86	1332.741	LELQEVVDFLK	2	3.1186		
					5	85	1524.748	SYLENQM(+15.995)AVALGGR	2	3.0634		
Serial#	Gi	Protein Name	Comment	Identified peptides								Coverage
3	7525018	ATP synthase CF1 alpha subunit [Arabidopsis thaliana]		4								9%
						> Score	> Mass	Sequence	Charge	xCorr		
					1	82	876.5191	FLLQEKV	2	1.6795	*	
					2	90	1252.701	VINALANPIDGR	2	3.5935		
					3	85	1252.726	LIESPAPGIISR	2	2.849		
					4	89	1416.785	IAQIPVSEAYLGR	2	2.6379		
Serial#	Gi	Protein Name	Comment	Identified peptides								Coverage
4	7525040	ATP synthase CF1 beta subunit [Arabidopsis thaliana]		3								8%
						> Score	> Mass	Sequence	Charge	xCorr		
					1	86	975.5624	IGLFGGAGVGK	2	2.9443		
					2	85	1415.713	TNPTTSNPEVSIR	2	1.4419	*	
					3	80	1517.744	ESGVINEQNLAESK	2	2.5698		
Serial#	Gi	Protein Name	Comment	Identified peptides								Coverage
5	7525033	photosystem I P700 chlorophyll a apoprotein A1 [Arabidopsis thaliana]		2								2%
						> Score	> Mass	Sequence	Charge	xCorr		
					1	76 76	880.3796	DYDPTNR	2	1.1627	*	
					2	81	1062.526	YSEFLTFR	2	2.1231	*	
Serial#	Gi	Protein Name	Comment	Identified peptides								Coverage
6	15232249	LHCB2:4 (Photosystem II light harvesting complex gene 2.3); chlorophyll binding [Arabidopsis thalian ...		2								7%
	18397286	CAB3 (CHLOROPHYLL A/B BINDING PROTEIN 3); chlorophyll binding [Arabidopsis thaliana]										7%
	16374	chlorophyll a/b binding protein (LHCP AB 180) [Arabidopsis thaliana]										8%
	15220615	CAB1 (CHLOROPHYLL A/B BINDING PROTEIN 1); chlorophyll binding [Arabidopsis thaliana]										7%
	18403546	LHB1B2 (Photosystem II light harvesting complex gene 1.5); chlorophyll binding [Arabidopsis thaliana ...										7%
	18403549	LHB1B1 (Photosystem II light harvesting complex gene 1.4); chlorophyll binding [Arabidopsis thaliana ...										7%
	15224465	LHCB2.2 (Photosystem II light harvesting complex gene 2.2); chlorophyll binding [Arabidopsis thalian ...										7%
	15224471	LHCB2.1 (Photosystem II light harvesting complex gene 2.1); chlorophyll binding [Arabidopsis thalian ...										7%
	4741944	Lhcb2 protein [Arabidopsis thaliana]										7%
	12324161	chlorophyll A-B-binding protein 2 precursor, 5 _quote_ partial; 1-750 [Arabidopsis thaliana]										7%
	4741948	Lhcb2 protein [Arabidopsis thaliana]										7%
	17473828	chlorophyll a/b-binding protein [Arabidopsis thaliana]				> Score	> Mass	Sequence	Charge	xCorr		7%
					1	89	1252.676	NRELEVIHSR	3	3.7774		
					2	76	983.4987	FGEAVWFK	2	2.9319		
Serial#	Gi	Protein Name	Comment	Identified peptides								Coverage
7	7525041	ribulose-1,5-bisphosphate carboxylase/oxygenase large subunit [Arabidopsis thaliana]		2								4%
	1944432	ribulosebisphosphate carboxylase [Arabidopsis thaliana]										5%
	27752799	ribulose 1,5-bisphosphate carboxylase/oxygenase large chain [Arabidopsis thaliana]										5%

	27752813	ribulose 1,5-bisphosphate carboxylase/oxygenase large chain [Arabidopsis lyrata subsp. lyrata]				> Score	> Mass	Sequence	Charge	xCorr	5%
					1	83	1021.531	DTDILAAFR	2	2.7963	
					2	93	1228.653	DLAVEGNEIIR	2	3.0323	
Serial#	Gi	Protein Name	Comment	Identified peptides							Coverage
8	7525029	photosystem II 44 kDa protein [Arabidopsis thaliana]		1							3%
	27435858	photosystem II CP43 protein [Arabidopsis thaliana]									4%
	172045852	RecName: Full=Photosystem II 44 kDa reaction center protein; AltName: Full=P6 protein; AltName: Full ...				> Score	> Mass	Sequence	Charge	xCorr	3%
					1	94	1426.765	LGANVGSAQGPTGLGK	2	3.3656	

Seq 20189 - #3

Serial#	Gi	Protein Name	Comment	Identified peptides							Coverage
1	7525018	ATP synthase CF1 alpha subunit [Arabidopsis thaliana]		5							11%
						> Score	> Mass	Sequence	Charge	xCorr	
					1	82	876.5191	FLLQEKV	2	1.6795	
					2	80	1604.865	TNKPQFQEIIASTK	3	3.1529	
					3	90	1252.701	VINALANPIDGR	2	3.5935	
					4	85	1252.726	LIESPAPGIISR	2	2.849	
					5	89	1416.785	IAQIPVSEAYLGR	2	2.6379	
Serial#	Gi	Protein Name	Comment	Identified peptides							Coverage
2	7525033	photosystem I P700 chlorophyll a apoprotein A1 [Arabidopsis thaliana]		2							2%
						> Score	> Mass	Sequence	Charge	xCorr	
					1	76 76	880.3796	DYDPTNR	2	1.1627	
					2	81	1062.526	YSEFLTFR	2	2.1231	
Serial#	Gi	Protein Name	Comment	Identified peptides							Coverage
3	15232249	LHCB2:4 (Photosystem II light harvesting complex gene 2.3); chlorophyll binding [Arabidopsis thalian ...		2							7%
	18397286	CAB3 (CHLOROPHYLL A/B BINDING PROTEIN 3); chlorophyll binding [Arabidopsis thaliana]									7%
	16374	chlorophyll a/b binding protein (LHCP AB 180) [Arabidopsis thaliana]									8%
	15220615	CAB1 (CHLOROPHYLL A/B BINDING PROTEIN 1); chlorophyll binding [Arabidopsis thaliana]									7%
	18403546	LHB1B2 (Photosystem II light harvesting complex gene 1.5); chlorophyll binding [Arabidopsis thaliana ...									7%
	18403549	LHB1B1 (Photosystem II light harvesting complex gene 1.4); chlorophyll binding [Arabidopsis thaliana ...									7%
	15224465	LHCB2.2 (Photosystem II light harvesting complex gene 2.2); chlorophyll binding [Arabidopsis thalian ...									7%
	15224471	LHCB2.1 (Photosystem II light harvesting complex gene 2.1); chlorophyll binding [Arabidopsis thalian ...									7%
	4741944	Lhcb2 protein [Arabidopsis thaliana]									7%
	12324161	chlorophyll A-B-binding protein 2 precursor, 5 _quote_ partial; 1-750 [Arabidopsis thaliana]									7%
	4741948	Lhcb2 protein [Arabidopsis thaliana]									7%
	17473828	chlorophyll a/b-binding protein [Arabidopsis thaliana]				> Score	> Mass	Sequence	Charge	xCorr	7%
					1	89	1252.676	NRELEVIHSR	3	3.7774	
					2	76	983.4987	FGEAVWFK	2	2.9319	
Serial#	Gi	Protein Name	Comment	Identified peptides							Coverage
4	7525041	ribulose-1,5-bisphosphate carboxylase/oxygenase large subunit [Arabidopsis thaliana]		1							2%
	1944432	ribulosebisphosphate carboxylase [Arabidopsis thaliana]									2%
	27752799	ribulose 1,5-bisphosphate carboxylase/oxygenase large chain [Arabidopsis thaliana]									2%
	27752813	ribulose 1,5-bisphosphate carboxylase/oxygenase large chain [Arabidopsis lyrata subsp. lyrata]				> Score	> Mass	Sequence	Charge	xCorr	2%
					1	83	1021.531	DTDILAAFR	2	2.7963	

Seq 20190 - #4 - No Identification

Seq 20191 - #5 - No Identification
